# Synthesis, Characterization and Host-Guest Complexation of Asplatin: Improved In Vitro Cytotoxicity and Biocompatibility as Compared to Cisplatin

**DOI:** 10.3390/ph15020259

**Published:** 2022-02-21

**Authors:** Sherif Ashraf Fahmy, Fortuna Ponte, Giulia Grande, Iten M. Fawzy, Asmaa A. Mandour, Emilia Sicilia, Hassan Mohamed El-Said Azzazy

**Affiliations:** 1 Department of Chemistry, School of Sciences & Engineering, The American University in Cairo, AUC Avenue, P.O. Box 74, New Cairo 11835, Egypt; sheriffahmy@aucegypt.edu; 2 School of Life and Medical Sciences, University of Hertfordshire Hosted by Global Academic Foundation, R5 New Garden City, New Administrative Capital, AL109AB Cairo 11835, Egypt; 3 Department of Chemistry and Chemical Technologies, University of Calabria, Arcavacata di Rende 87036, Italy; fortuna.ponte@unical.it (F.P.); giulia-grande@hotmail.com (G.G.); emilia.sicilia@unical.it (E.S.); 4 Pharmaceutical Chemistry Department, Faculty of Pharmacy, Future University in Egypt, Cairo 11835, Egypt; iten.mamdouh@fue.edu.eg (I.M.F.); asmaa.abdelkereim@fue.edu.eg (A.A.M.)

**Keywords:** cisplatin, acetylsalicylic acid, asplatin, platinum(IV) prodrugs, *p*-sulfocalix[4]arenes, host-guest complexation, DFT, cancer therapy

## Abstract

*Para*-sulfocalix[n]arenes are promising host molecules that can accommodate various chemotherapeutic drugs. Pt(IV)-based complexes, including satraplatin and asplatin, are promising alternatives that overcome the shortcomings of Pt(II) complexes. In this study, asplatin has been synthesized by fusing acetylsalicylic acid (aspirin) and cisplatin. Furthermore, it has been characterized using ^1^H NMR, mass spectrometry, elemental analysis, and UHPLC. A host-guest complex of asplatin and *p*-sulfocalix[4]arene (PSC4) has been developed and characterized using UV, Job’s plot analysis, HPLC, and density functional theory (DFT) calculations. The experimental and computational investigations propose that a 1:1 complex between asplatin and PSC4 is formed. The stability constant of the designed complex has been determined using Job’s plot and UHPLC and computed to be 9.1 × 10^4^ M^–1^ and 8.7 × 10^4^ M^−1^, which corresponds to a free energy of complexation of −6.8 kcal mol^–1^, while the calculated value for the inclusion free energy is −13.2 kcal mol^−1^. Both experimentally and theoretically estimated complexation free energy show that a stable host-guest complex can be formed in solution. The in vitro drug release study displayed the ability of the complex to release its cargo at a cancerous pH (pH of 5.5). Additionally, the asplatin/PSC4 complex is shown to be biocompatible when tested on human skin fibroblast noncancerous cells, demonstrating the highest in vitro cytotoxic activity against (MCF-7), cervical (HeLa), and lung cancer cells (A-549), with IC_50_ values of 0.75, 2.15, and 3.60 µg/mL, respectively. This is as compared to either cisplatin (IC_50_ of 5.47, 5.94 and 9.61 µg/mL, respectively) or asplatin (IC_50_ of 1.54, 5.05 and 3.91 µg/mL, respectively). On the other hand, the free asplatin exhibited higher cytotoxicity on cancerous cells and lower toxicity on noncancerous cells. The outcomes of the present joint theoretical and experimental investigation reinforce the interest in platinum-based anticancer therapeutics when they are protected from undesired interactions and suggest the use of the PSC4 macromolecule as a promising carrier for Pt(IV) anticancer drugs. The formed asplatin/PSC4 inclusion complex may represent an effective chemotherapeutic agent.

## 1. Introduction

Platinum-based anticancer drugs (PBDs) are powerful broad-spectrum antitumor treatments effective against many solid tumors, including breast cancer. Cisplatin (cis-diammine-(dichlorido)platinum(II)) is a first-generation platinum II-based complex that was granted US Food and Drug Administration (FDA) approval in the late 1970s [1,2,3,4]. To date, hundreds of PBDs have been synthesized and tested in clinical trials to enhance their anticancer activities, minimize toxic effects (including nephrotoxicity and emetogenicity), and overcome resistance as compared to cisplatin. Among these developed PBDs, only two Pt(II)-based drugs, carboplatin and oxaliplatin, have been approved by the FDA for cancer therapy [5]. However, Pt(II)-based anticancer drugs still suffer many drawbacks because of their chemical reactivity, including the inconvenient intravenous route of administration, systemic adverse reactions, and acquired and/or intrinsic treatment resistance. On the contrary, Pt(IV)-based drugs are promising substitutes that overcome the shortcomings of Pt(II) complexes. This is because they are kinetically more inert, and consequently, they could be administered via the oral route and demonstrate lower systemic toxicity than Pt(II) complexes [4]. Pt(IV) complexes act as prodrugs that, upon cellular entry, are activated by cellular reducing agents (such as ascorbic acid and glutathione), forming the corresponding Pt(II) active species. Many efforts have been devoted to the elucidation of the Pt(IV) reduction mechanisms from both theoretical [6,7] and experimental [8,9] points of view over the years. Satraplatin (trans,cis,cis-bis(acetato)ammine-cyclohexylaminedichloridoplatinum(IV)) is the first designed oral Pt(IV)-based anticancer drug that is presently in phase III clinical trials in curing prostatic cancer [4]. Recently, the new dual-action Pt(IV) complex asplatin, c,c,t-[PtCl_2_(NH_3_)_2_(OH)(acetylsalicylic acid)], has been synthesized by tethering acetylsalicylic acid (aspirin) to the Pt center of cisplatin. Tests have proved asplatin to exhibit more potent anticancer activity and lower adverse effects when compared to cisplatin [10]. The anticancer activity of acetylsalicylic acid (ASA) and its metal complex derivatives (such as ASA-derived chlorinated cobalt alkyne complexes) against many types of cancers has been reported [11]. Furthermore, computational studies of the activation mechanism of the asplatin prodrug have been recently carried out by means of density functional theory (DFT) [12] and the multi-class harmonic linear discriminant analysis (MC-HLDA)-metadynamic method [13]. Nevertheless, Pt(IV) complexes still have shortcomings that hinder their clinical applications. For instance, Pt(IV) complexes might suffer a premature reduction in the systemic circulation before reaching cancer cells, leading to diminished bioavailability and side effects [14,15]. Additionally, tetraplatin Pt(IV) complex has been shown to cause severe neurotoxicity, and, as a consequence, its clinical application has been abandoned [15]. Hence, PBDs have been reformulated into various delivery platforms that could advance their selective uptake into target cancer cells while concurrently minimizing undesired toxic effects. Several delivery vehicles, such as liposomes, solid lipid nanoparticles, polymeric nanoparticles, and supramolecular host molecules, have been reported to encapsulate PBDs and other therapeutic agents [16,17,18,19,20,21].

Supramolecular host molecules, comprising calix[n]arenes (CXs), cyclodextrins (CDs), cucurbiturils (CBs), and pillararenes, have been suggested as smart drug delivery platforms [1,22,23] and have been used as host molecules to encapsulate various chemotherapeutic drugs to improve their bioavailability, prevent their premature degradation in the bloodstream, and increase their targeted intracellular uptake into cancer cells [1,2,3]. Lately, calix[n]arenes (*n* = 4, 6, and 8) have been employed in drug targeting [24,25,26]. Calixarenes are idyllic host molecules that are synthesized by linking phenolic rings via methylene bridges encompassing three regions; (1) a lower rim with a phenolic hydroxyl group, (2) an upper rim with a *para*-substituent of a phenolic unit, and (3) a hydrophobic π electron-rich central cavity. Sulfonate groups are utilized to functionalize the water-insoluble calixarenes through their phenolic rings’ *para*-positions, generating *para*-sulfonatocalix[n]arenes [5,20]. *Para*-sulfonatocalix[n]arenes (PSC4) are water-soluble (>0.1 mol L^−1^) and innocuous to human cells at doses up to 10^9^ ng/kg [1]. Moreover, *para*-sulfonatocalix[n]arenes can accommodate numerous guest molecules in their cavities via the host-guest inclusion complexation [1].

In this work, asplatin has been synthesized and characterized using ^1^H NMR spectroscopy and mass spectrometry. Furthermore, a novel UHPLC method has been developed for detecting asplatin. Then, complexation between *Para*-sulfonatocalix[n]arenes (PSC4) and asplatin in an aqueous medium has been investigated for possible application in cancer therapy. The developed complex has been characterized by UV, UHPLC, and computational studies. The stoichiometry and binding constants have been determined by Job’s plot (continuous variation method) and quantum mechanical calculations at the density functional theory (DFT) level. Theoretically, the structural characterization of the formed host-guest complexes has been carried out. Intermolecular hydrogen bonds in the asplatin/PSC4 adducts have been studied by means of the atoms in molecules (AIM) theory. Moreover, the cytotoxic activities of the complex against breast adenocarcinoma (MCF-7), cervical cancer (HeLa), and lung cancer (A549) have been studied and compared to free asplatin and cisplatin. The biocompatibility of the designed complex has also been evaluated using human skin fibroblast noncancerous cells and compared to free asplatin and cisplatin.

## 2. Results and Discussion

### 2.1. Synthesis and Characterization of Asplatin

Asplatin has been synthesized as reported previously with some modifications [27,28].

**Scheme 1:**
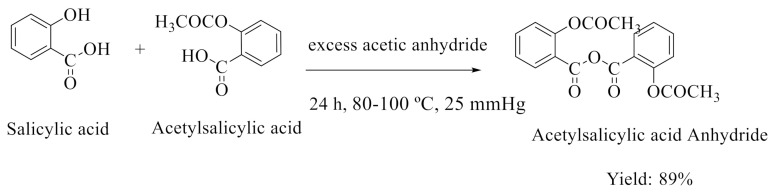
Synthesis of acetylsalicylic acid anhydride.

**Scheme 2:**
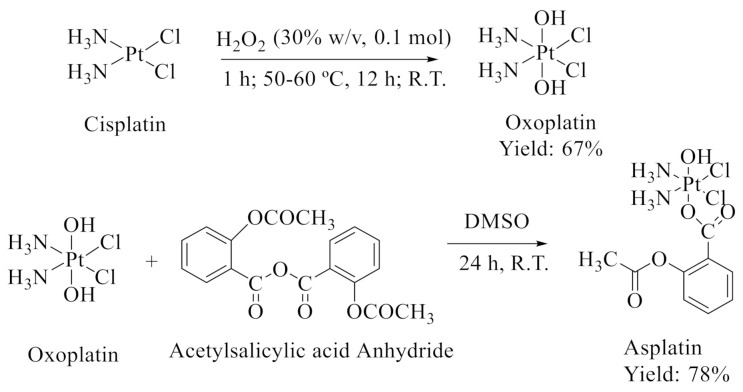
Synthesis of asplatin.

The synthetic pathways of acetylsalicylic acid anhydride and asplatin are presented in Scheme 1 and Scheme 2, respectively.

**Figure 1:**
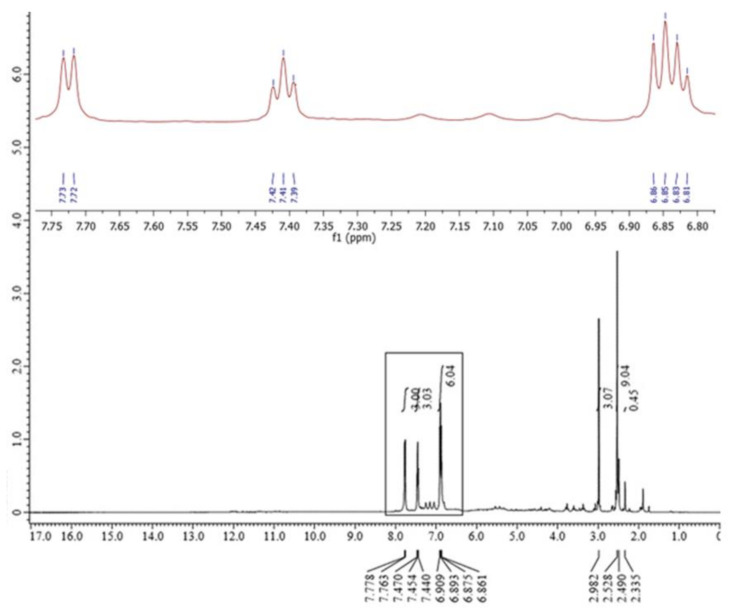
^1^H-NMR characterization of asplatin.

The formation of asplatin has been confirmed using ^1^H-NMR spectroscopy (400 MHz, DMSO-d6), as presented in Figure 1. The aromatic phenyl hydrogens of asplatin appeared as two doublets and two triplets in the range of δ 7.77–6.86 ppm, while the aliphatic methyl hydrogens appeared at δ 2.52 ppm, indicating the attachment of the acetyl group to the aromatic ring. Different singlet signals appeared at δ 2.33, 2.49 and 2.98 ppm, indicating the D_2_O-exchangeable signals of a hydroxyl and two amine groups, respectively.

The melting point for asplatin was 139–141 °C. Furthermore, mass spectrometry and elemental analysis were used to confirm the synthesis of asplatin (Table 1).

Furthermore, ^1^H-NMR was used to confirm the formation of a 1:1 M ratio asplatin/PSC4 complex (Figure S1, Supplemetary Materials). The aromatic phenyl hydrogens appeared as one doublet, one triplet and multiplets in the range of δ 7.75–6.85 ppm, and the aromatic hydrogens of PSC4 appeared as multiplets in the range of δ 6.87–6.82 ppm. The hydrogens of PSC4 methylene groups showed multiplet signals at δ 3.34 ppm, while the aliphatic hydrogens of the asplatin methyl group appeared as a singlet at δ 2.49 ppm. Finally, dispersed signals appeared in the spectrum at δ 12.48, 6.0–5.80, 3.34 and 2.52 ppm, indicating the D_2_O exchangeable signals of PSC4 hydroxyl groups and asplatin amine groups. From the above data, the signals representing hydrogens of both asplatin and PSC4 coexist in the ^1^H-NMR performed for the complex. Additionally, the disappearance of asplatin’s OH signal (at 2.33 ppm) may suggest hydrogen bonding within the host.

**Figure 2:**
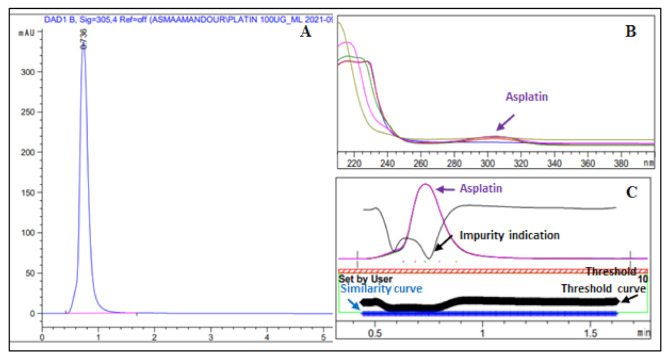
(**A**) UHPLC chromatogram of asplatin 100 µg/mL (0.2 mM), retention time of 0.736 min. (**B**) UV Spectrum at λ max 305 nm showing similarity of absorbances of asplatin (purple line) containing impurities with a purity factor of about 900 showing spectral similarity. (**C**) Similarity (blue) and threshold (black) curves show asplatin’s impurity content within the threshold limit (in the green band).

Analysis of the synthesized asplatin using UHPLC showed a sharp symmetric peak at a retention time of 0.736 min (Figure 2A). Measurement has been performed at a λ max of 305 nm (Figure 2B). The sample showed good purity as indicated by a threshold limit of 10 (Figure 2C). Peak purity, measured as the ratio compared to the threshold, was automatically determined using DAD by evaluating the spectral homogeneity across the peak. The UHPLC report showed a purity factor close to 900 within the threshold limit (represented by the broken line and indicating the range for spectral impurity within the noise limit). The intersection between the similarity curve with the threshold curve lies above the threshold limit, indicating an impurity (Figure 2B). The similarity curve exhibits a very similar profile within the threshold limit, and the peak purity ratio is clear within the green band, indicating the good purity of asplatin (Figure 2C).

### 2.2. Asplatin/PSC4: UV-Vis Spectroscopy Analysis

The host-guest inclusion complexation between asplatin and PSC4 in an aqueous medium has been studied applying UV-Vis spectroscopy [16].

**Figure 3:**
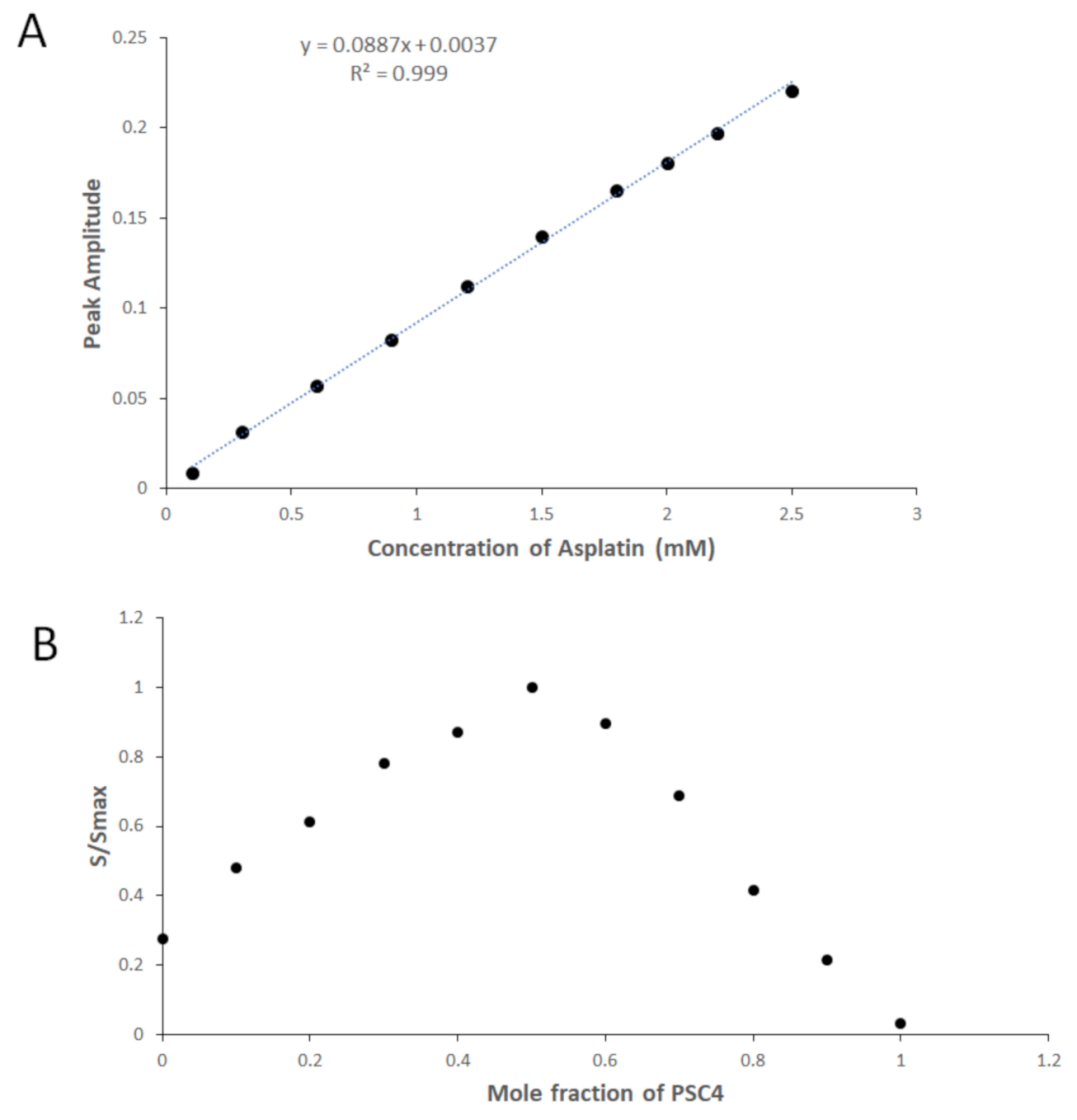
(**A**) Absorbance spectra of 0.2 mM PSC4 and various mixtures comprised consecutively of increasing concentrations of asplatin (ranging from 0.00–0.25 mM) and a fixed concentration of 0.2 mM PSC4 all in aqueous media. (**B**) Normalized Job’s plot for asplatin/PSC4 inclusion complex. Measurements were carried out in triplicates.

The UV absorbance spectra of mixtures of 0.2 mM PSC4 and increasing asplatin concentrations (ranging from 0.01–0.25 mM) have been recorded in aqueous media (Figure S2, Supplementary Materials). The spectrum of PSC4 exhibited apparent absorption maxima at 277 and 284 nm (Figure S2). The spectra of the mixtures demonstrated a remarkable hyperchromic shift, which has been interrelated with increasing asplatin concentration in the range of 0.01–0.25 mM. Furthermore, increasing asplatin concentration in the mixture has been accompanied by a gradual merging of the typical pair of absorption maxima for PSC4 and the generation of a new individual maxima at 281 nm. This suggests the formation of a host-guest inclusion complex between asplatin and PSC4, as reported previously [16]. To investigate the hyperchromic shift that should occur with increasing concentrations of asplatin in the prepared mixtures, the PSC4 spectrum has been used as a divisor for the zero-order spectra of the mixtures. The first derivative of the ratio spectra has been calculated using a scaling factor of 10 and Δλ = 8 nm [16]. The peak amplitudes’ values of the first derivative of the ratio spectra for the mixtures have been then found at 264 nm. Figure 3A depicts a plot of the obtained peak amplitudes and the subsequent concentrations of asplatin. The ultimate linear relationships presented in Figure 3A indicated that the hyperchromic shifts noted in asplatin/PSC4 are due to a host-guest complex formation between PSC4 and asplatin.

The stoichiometry and the prepared complex’s stability constants have been studied using the method of continuous variation (Job’s plot) involving the derivative ratio method as reported previously [16]. Job’s plot showed that the maximum amplitude had been noticed at a molar fraction of 0.5, proposing a host-guest inclusion complex stoichiometry of 1:1 asplatin/PSC4. Then, each amplitude value, S, was divided by the equivalent maximum amplitude, Smax, forming the normalized form of Job’s plot (Figure 3B). The stability constant of the asplatin/PSC4 inclusion complex found utilizing our previously reported method [16] was 9.1 × 104 M^−1^, corresponding to a complexation free energy of −6.8 kcal mol^−1^. This value is within the stability constant range (0.001 × 10^4^ to 16 × 10^4^ M^–1^) of host-guest complexes designed for drug delivery applications [29,30,31,32,33,34,35,36,37,38,39,40].

### 2.3. Asplatin/PSC4: UHPLC Analysis

A UHPLC diode array has been utilized to study the complexation between asplatin and PSC4. A photodiode array detector enabled the simultaneous determination of PSC4 at 267 nm and asplatin at 305 nm. At a fixed concentration of asplatin, a sharp peak has been shown to lessen upon increasing the concentration of PSC4, which suggests the formation of the asplatin/PSC4 complex.

**Figure 4:**
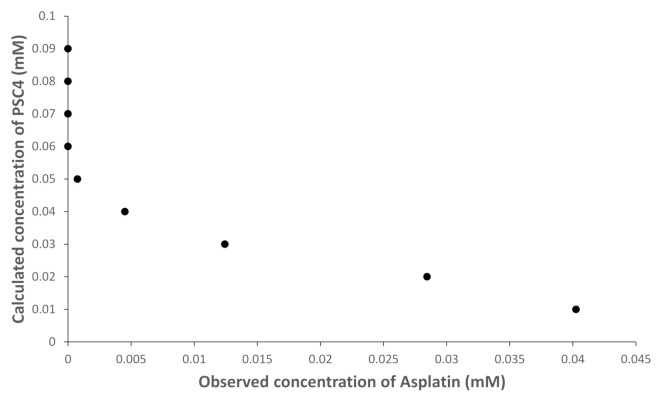
UHPLC observed concentrations of asplatin of solutions containing a fixed concentration of asplatin (0.05 mM) and varying concentrations of PSC4 (0.01–0.09).

Consequently, we tested solutions of changing molar ratios formed of a fixed concentration of asplatin (0.05 mM) and varying concentrations of PSC4 (0.01–0.09 mM). As presented in Figure 4, asplatin (at a concentration of 0.05 mM) was not detected when PSC4 was in the range of 0.09–0.06 mM. This proposed that at these molar ratios, almost all the asplatin in these solutions has been complexed with PSC4. Furthermore, Figure 4 shows a signal for asplatin to be detected firstly only when equal molar ratios of 0.05 mM for each asplatin and PSC6 existed in the medium, suggesting a 1:1 asplatin to PSC4 host-guest inclusion complexation, as presented previously. The stability constant of asplatin/PSC4 has been computed to be 8.7 × 10^4^ M^−1^, which is close to the stability constant computed from Job’s plot.

### 2.4. In Vitro Release Study

**Figure 5:**
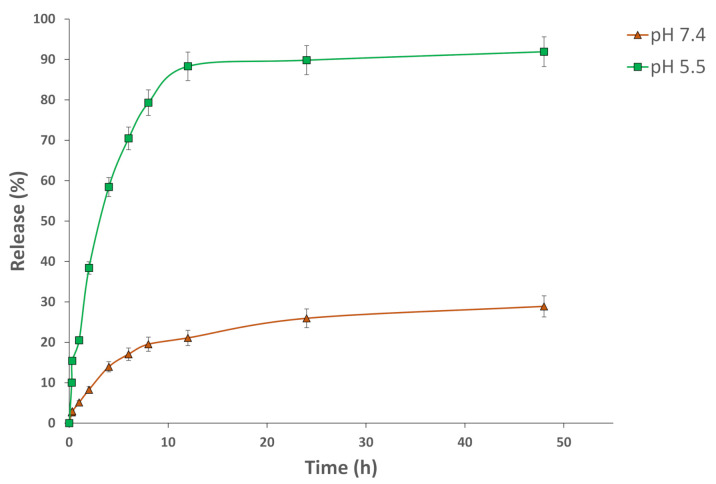
Time-dependent release profiles of asplatin at 37 °C, into pH 5.5 (square) and pH 7.4 (triangle) in phosphate buffer media.

The release of asplatin from the asplatin/PSC4 complex was investigated at 37 °C and pH 7.4 (of noncancerous cells) and pH 5.5 (of cancer cells) using the dialysis bag method (Figure 5). The released asplatin was quantified utilizing the UHPLC method described below. The developed host-guest complex displayed outstanding stability at the physiological pH, with about 28.9% of the included asplatin released after 48 h. At the cancerous cell pH (pH 5.5), 92.0% of the incorporated drug was released after 48 h. These findings suggest the ability of the designed complex to release its cargo selectively in cancerous cells via a pH-triggered mechanism. In the acidic medium, the host-guest complex is disassembled, leading to the drug release from the complex [41]. Thus, the asplatin/PSC4 complex is a promising system that could be applied in future cancer remedies because of its ability to release the incorporated drugs inside the cancer tissue while protecting them from premature degradation in the physiological pH of systemic circulation.

### 2.5. In Vitro Cell Viability Assay

The cytotoxicity of PSC4, cisplatin, asplatin, and asplatin/PSC4 complex was evaluated on normal human skin fibroblasts, breast adenocarcinoma cells (MCF-7), cervical cancer cells (HeLa), and lung cancer cells (A-549) utilizing an SRB assay (Table 2 and Figure S3, Supplementary Materials). PSC46 has been employed as a host control, and it had no remarkable cytotoxicity on both cancer and noncancerous cells. The cytotoxicities of free and complexed asplatin were tested on normal and cancer cells and compared to cisplatin (positive control). After 48 h incubation, the asplatin/PSC4 complex showed the highest in vitro cytotoxic activity against MCF-7, HeLa, and A-549 cancer cells, with IC_50_ values of 0.75, 2.15, and 3.60 µg/mL, respectively; this is as compared to either cisplatin (IC_50_ of 5.47, 5.94 and 9.61 µg/mL, respectively) and asplatin (IC_50_ of 1.54, 5.05 and 3.91 µg/mL, respectively). Additionally, it has been shown that asplatin/PSC4 exerted higher cytotoxic activity against the three cancer cells as compared to asplatin. On the other hand, asplatin showed lower cytotoxicity against human skin fibroblast cells than cisplatin (IC_50_ of 3.41 and 1.20 µg/mL, respectively). Pt(IV)-based drugs serve as prodrugs that, upon cellular entry, are reduced and activated by the cellular reductants (ascorbic acid and glutathione), forming the Pt(II) active candidate. Pt(II)-activated species exert their anticancer effects via interacting with DNA, forming Pt-DNA adducts [4]. Overall, the prepared asplatin/PSC4 host-guest complex exhibited improved anticancer activity on cancerous cells while maintaining minimum cytotoxic activity against noncancerous cells as compared to free asplatin (Table 2). On the other hand, calixarenes are pH sensitive and could release their cargo selectively in the acidic cancer cell microenvironment [5,16,17,41]. Hence, developing the asplatin/PSC4 complex improves the drug targeting of tumor cells (pH of 5.5) while minimizing off-target toxic effects.

It is worth mentioning that the asplatin/PSC4 complex displayed stronger anticancer activities against MCF-7 as compared to our previously prepared PSC4 complexes incorporating Pt(II)-based drugs, oxaliplatin/PSC4 complex (IC50 of 1.56 µg/mL) [5] and carboplatin/PSC4 (IC50 of 4.3 µg/mL) (Table 3) [3]. Additionally, the asplatin/PSC4 complex showed a more potent anticancer activity than the carboplatin/PSC4 complex against the A-549 cancer cell line (IC50 of 5 µg/mL) (Table 3) [3].

### 2.6. Computational Analysis of H-G Adducts

**Figure 6:**
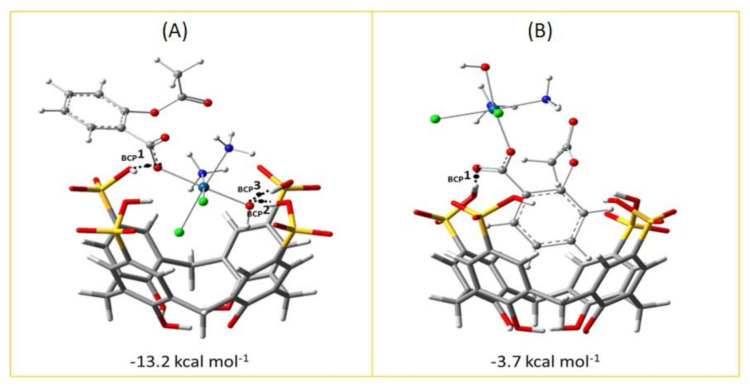
B97-D optimized geometry of the two most stable asplatin/PSC4 inclusion complexes along with the characterized BCPs and the free energy of inclusion values (kcal mol^−1^). (**A**) The platinum center is included in the PSC4 cavity with the phenyl ring of the aspirinate ligand outside the larger rim. (**B**) The platinum center is located outside the PSC4 cavity, with the phenyl ring included within.

DFT calculations have been carried out to simulate the inclusion of asplatin into PSC4 calixarene to form the corresponding host-guest inclusion complex. The fully optimized geometries obtained without imposing any constraints are reported in Figure 6, as are the calculated inclusion Gibbs free energy values, including BSSE and entropy change corrections in solution.

The free energies of inclusion have been evaluated as the difference between the free energy of the inclusion adduct and the sum of the asplatin (host) and PSC4 (guest) free energies for two different conformations: (a) the conformation of minimum energy in which the platinum center is included inside the host cavity and the aspirinate ligand, specifically its phenyl ring, points outward establishing minimal interactions with the host, and (b) the phenyl ring is positioned in the center of the host cavity, and the acetyloxy moiety and rest of the platinum complex points outward. In both obtained inclusion complexes, the axial ligands play an important role in stabilizing the supramolecular system forming hydrogen bonds with the SO_3_H groups, decorating the upper rim of the host cavity.

The results reported in Figure 6 indicate that the insertion process of asplatin in the PSC4 macromolecule is thermodynamically favorable and suggests a good propensity of the host to maximize interactions with this guest. The guest molecule approaches the host from the upper rim of the PSC4 macromolecule. In particular, for the host-guest complex in the (a) conformation, the most stable adduct is 13.2 kcal mol^−1^, which is more stable with respect to the fully minimized separated species. Instead, the host-guest adduct in the (b) conformation is less stable than (a), with an inclusion energy of −3.7 kcal mol^−1^, which implies a lower propensity of the host to adapt its cavity when the platinum complex is rotated with the aromatic ring pointing inside it. The minimum energy structure characteristics of the (a) conformation agree with the experimental data.

With the aim to characterize in more depth the nature of the host-guest interactions for both the intercepted asplatin-PSC4 complexes, AIM analysis has been performed. The nature and the strength of the interactions have been evaluated following the Bader and Essén theory through the value of the electronic density at the Bond Critical Point (*ρ*BCP) and its Laplacian (∇^2^*ρ*BCP) [42]. Specifically, for non-covalent interactions such as van der Waals and hydrogen bonding, *ρ*(*r*) is relatively small. In particular, the value of *ρ*(*r*) is ∼10^−3^ a.u. for Van der Waals interactions and ∼10^−2^ a.u. or less for H-bonds, and the corresponding Laplacian ∇^2^*ρ*(*r*), in both the cases, is positive.

The electron density and the Laplacian values calculated at the BCPs are reported in Figure 6. For both the insertion complexes, the values calculated for the electron density values fall within the range 0.03–0.08 a.u., and the sign of the Laplacian is positive, highlighting the possible formation of hydrogen bonds which play an important role in stabilizing the investigated systems.

The most stable complex with asplatin in the (a) configuration presents three critical points indicated as _BCP_**1**, _BCP_**2,** and _BCP_**3**. The values of the Laplacian of *ρ* at the (3, −1) BPCs for the hydrogen bonds linking the asplatin to the PSC4 are 0.084, 0.151, and 0.145 a.u., respectively. The strength of these hydrogen bonds in the (a) adduct is verified by the high positive values of the Laplacian. The hydrogen bond lengths correlating to _BCP_**1**, _BCP_**2** and _BCP_**3** are equal to 1.852, 1.477, and 1.514 Å, respectively. Therefore, in the system examined here, the synergistic effect of the hydrogen bonding interactions plays a central role in determining the stability of this host-guest inclusion complex.

In the host-guest complex (b) shown in Figure 6, in which the phenyl ring of the asplatin complex is located inside the macrocycle, the electron density value for the _BCP_**1** is 0.083. The value of the Laplacian is positive, and the hydrogen bond length is very short and is equal to 1.461 Å. Here, however, the presence of the aromatic ring in the structure of the Pt chemotherapeutic agent contributes to the stabilization of the inclusion complex through hydrophobic interactions. The host-guest hydrophobic interactions, together with the hydrogen bond mentioned above, established between the carboxylate group directly bound to the platinum center and the SO_3_H group characterize the inclusion complex.

## 3. Experimental and Computational Details

### 3.1. Chemicals and Reagents

*Para*-Sulfocalix[4]arene, PSC4, was purchased from BLD Pharmatech Company (Cincinnati, OH, USA). Streptomycin, penicillin, fetal bovine serum, trichloroacetic acid (TCA), Dulbecco’s Modified Eagle’s Medium (DMEM) SRB, and tris(hydroxymethyl)aminomethane (TRIS) were purchased from Lonza, Basel, Switzerland. All other reagents have been obtained from Sigma Aldrich (St. Louis, MO, USA).

### 3.2. Instrumentation

The ^1^H-NMR analysis was carried out using a JEOL type GSX-270 or JNM-ECA 500II spectrometer (Jeol, Peabody, MA, USA). Mass spectrometry was conducted on a Shimadzu GCMS-QP 5050A gas chromatograph-mass spectrometer. HPLC measurements were carried out using an Agilent ultra-performance 1290 infinity, equipped with 1290 DAD, a gradient quaternary pump VL, an auto-sampler ALS, a column oven TCC and a 1290 Thermostat. Data acquisition was performed using Agilent Chemstation software (B.04.03), and data processing was subsequently performed using Agilent LabAdvisor (Utility) Quantitative analysis software (B.02.04). UV spectrophotometric measurements were carried out on a CARY 500 UV–Vis-NIR Scan dual-beam spectrophotometer (Varian, Palo Alto, CA, USA).

### 3.3. Synthesis of Asplatin

The synthesis of asplatin was conducted as reported elsewhere, with some modifications [27,28]. First, acetylsalicylic acid anhydride were synthesized, such that an equimolar amount of salicylic acid (0.014 mol, 2 g) and acetylsalicylic acid (0.014 mol, 2.5 g) were allowed to react under anhydrous conditions using acetic anhydride (0.028 mol, 2.85 g). The mixture was allowed to reflux for 1 h at a temperature range of 80 °C–100 °C, then maintaining a continually decreasing pressure over the ongoing reaction for 24 h to give a maximum absolute pressure of about 25 mmHg. The obtained product was then dissolved in an organic solvent such as dichloromethane and crystallized to get a pure yield of an acetylsalicylic acid anhydride.

Furthermore, oxoplatin was prepared via reacting 374 mg of cisplatin (1.23 mmol) suspended in 15 mL distilled water with 12.5 mL of H_2_O_2_ (30% *w*/*v*, 0.1 mol) added dropwise. The reaction was allowed to take place for 1 h at an increasing temperature from 50 °C to 100 °C; then, it was stirred for an extra 12 h at room temperature. The resultant was lyophilized, washed with cold water and ethanol and dried in a vacuum. Finally, asplatin was prepared by reacting a solution of oxoplatin (0.9 mmol, 300 mg) in 15 mL dimethylsulfoxide (DMSO) with the previously prepared acetylsalicylic acid anhydride (1.8 mmol, 620 mg), and the mixture was stirred for 24 h at room temperature. The obtained residue was washed with acetone and ether and dried under a vacuum. The final product of asplatin was collected as dark yellow crystals of yield 78% and characterized by different techniques.

The structural elucidation of asplatin was done by ^1^H-NMR (400 MHz, DMSO-d6) and showed the following signals: δ 7.77–7.76 (d, 1H, ArH), 7.47–7.44 (t, 1H, ArH), 6.90–6.89 (t, 1H, ArH), 6.87–6.86 (d, 1H, ArH), 2.98 (s, 3H, NH_3_), 2.52 (s, 3H, CH_3_), 2.49 (s, 3H, NH_3_) and 2.33 (s, 1H, OH).

### 3.4. Preparation of Asplatin/PSC4 Complex

The asplatin/PSC4 complex was prepared by mixing 0.2 mM PSC4 with different concentrations of asplatin (ranging from 0.01–0.25 mM) in aqueous media.

The structural elucidation of the asplatin/PSC4 complex by ^1^H-NMR showed the following signals: δ 12.48 (s, 1H, OH), 7.75–7.73 (d, 1H, ArH), 7.41–7.40 (t, 1H, ArH), 6.87–6.85 (m, 2H, ArH), 6.83–6.82 (m, 8H, ArH of PSC4), 6.0–5.80 (m, 4H, OH of PSC4), 3.34 (m, 8H, CH_2_ of PSC4 and m, 4H, OH of PSC4), 2.52 (s, 3H, NH_3_) and 2.49 (s, 3H, CH_3_).

The complex formation and stoichiometry were studied using UV-Vis Spectroscopy and UHPLC.

### 3.5. UHPLC Analysis

The synthesized asplatin and its complex with PSC4 were analyzed using a UHPLC diode array and a Zobrax Eclipse Plus C18 column (2.1 × 50 mm, 1.8 µm). The mobile phase consisted of acetonitrile: 0.1% orthophosphoric acid in the ratio of (80:20, *v/v*). The flow rate was 0.2 mL/min at a 30 °C column temperature with an injection volume of 1 µL.

### 3.6. In Vitro Release Study

The release percentage of asplatin from the asplatin/PSC4 complex was investigated using the dialysis bag method at pH 7.4 and 5.5. Concisely, 1 mL of the sample was inserted into a dialysis bag (cutoff molecular weight, 3500 Da). The dialysis bag was placed into 25 mL of PBS at pH 7.4 or 5.5. The whole system was placed in a shaking incubator (Jeio Tech SI-300, Seoul, Korea) and rotated at 100 rpm with a temperature maintained at 37 °C. At definite time intervals, a 1 mL aliquot of the sample was withdrawn for analysis by UHPLC and immediately replaced with another equal volume of warm buffer [17].

### 3.7. Cell Viability Assay

#### 3.7.1. Cell Culture

Breast adenocarcinoma cells (MCF-7), cervical cancer cells (HeLa), lung cancer cells (A-549), and human skin fibroblast healthy cells were obtained from American Type Culture Collection (University Boulevard, Manassas, VA, USA) and sustained in DMEM medium complemented with streptomycin (100 mg/mL), penicillin (100 units/mL), and 10% heat-inactivated fetal bovine serum. Cells were incubated in 5% (*v*/*v*) CO_2_ at 37 °C.

#### 3.7.2. Cell Viability Assay

Breast adenocarcinoma cells (MCF-7), cervical cancer cells (HeLa), lung cancer cells (A-549), and human skin fibroblast healthy cells were treated with various concentrations of PSC4, cisplatin (positive control), asplatin, and asplatin/PSC4 complex. The in vitro cytotoxic activities against either cancerous or noncancerous cells were evaluated utilizing Sulforhodamine B Colorimetric Assay (SRB assay), and the IC_50_ (in μg/mL) value was detected employing our previously reported methods [16,17,18]. Concisely, 100 μL cell suspension (5 × 10^3^ cells) was seeded in 96-well plates and incubated in DMEM medium for 24 h at 37 °C and 5% CO_2_. Cells were treated with medium (100 μL) containing different concentrations (from 0.01 to 300 μg/mL) of PSC4, cisplatin, asplatin, and asplatin/PSC4 complex. After 48 h, culture media were detached, and 10% TCA (150 μL) was added at 4 °C for 1 h. The cells were then washed several times with phosphate buffer saline. Then, 70 μL of SRB solution (0.4% *w/v*) was added and incubated in the dark for 10 min at room temperature. Acetic acid (1%) was used to wash the plates, and then the plates were left to dry for 24 h. Afterward, the protein-bound SRB stain was liquefied using 10 mM TRIS (150 μL), and absorbance was measured at 540 nm (FLUOstar Omega, Ortenberg, Germany) [16,17,18].

### 3.8. Computational Methods

All calculations were carried out at the DFT level of theory using the Gaussian16 package [43]. For the study of the inclusion systems, the B97-D exchange and correlation function [44] were adopted, and the investigated host-guest complexes were fully optimized in water media. Optimizations of the geometry were performed using the double-zeta 6-31G(d,p) basis set for all the involved atoms, except for oxygen atoms, for which the more extended 6-311+G(2df,2p) basis set was used, and for the platinum, for which the pseudo-potential SDD and its related valence basis set were employed [45].

The solvation effects were computed using the continuum solvation model based on density (SMD), [46] as implemented in Gaussian16, and setting a dielectric constant value of 80 corresponding to water as the solvent.

Frequency calculations within the harmonic approximation were also carried out at the same level of theory to explore and confirm the nature of the intercepted stationary points as minima and to calculate the Gibbs free energies for the inclusion of asplatin (G) into calix-4 arene (H).

The inclusion Gibbs free energy, in solution ∆Gsol, in implicit water, has been calculated as the difference between the free energy in solvent ($G_{sol}^{HG\;complex}$) of the H-G complex and the free energies of the separated species in solvent ($G_{sol}^{H}and\;G_{sol}^{G})$:

(1) $$∆G{\;}_{sol}^{HG\;complex}=\;G_{sol}^{HG\;complex}-\;\left(G_{sol}^{H}+G_{sol}^{G}\right)$$

The basis set superposition error (BSSE) estimated using the Boys–Bernardi counterpoise technique [47] was evaluated, and the binding energies were corrected, including this additional term.

The quantum theory of atoms in molecules (AIM) suggested by Bader [42] was used to characterize the molecular interactions responsible for the formation of the asplatin/PSC4 adducts. The methodology has been applied using the program package AIMAll [48] on the optimized structures.

The analysis of the electron density $\rho \left(r\right)$ around the region of the interaction allows the chemical bonding nature of the host-guest molecules to be identified. The values of $\rho \left(r\right)$ and its derivatives at the CPs (critical points, points where the gradient of the density is zero, $\nabla \rho \left(r\right)=0$) give information about the nature and the strength of interactions between H and G in molecular systems. To this aim, second-order derivatives of $\rho \left(r\right)\;\mathrm{have}\;\mathrm{to}\;\mathrm{be}\;\mathrm{explored},\;\mathrm{with}\;$ only nine derivatives attainable that are the elements relevant to generate a real and symmetric Hessian matrix of $\rho \left(r\right)$. The diagonalization of the matrix by a unitary transformation allows the corresponding eigenvalues $(\lambda_{n}$) to be found. The rank *ω* and the signature *σ* mark the CPs. The rank of the diagonalized matrix is given by the number of non-zero eigenvalues, namely non-zero curvatures of *ρ*(*r*) at the CPs. The signature of a CP, *σ,* is defined as the algebraic sum of the signs of eigenvalues (signs of curvatures of *ρ*(*r*) at the CPs). A CP with *ω* = 3 and *σ* = +1, corresponding to (3, +1) CP, has 2 positive curvatures, and *ρ* is a minimum at the CP in the plane defined by their corresponding axes. In contrast, at the CP along the third axis perpendicular to this plane, *ρ* is a maximum. The CP (3, −1), with *ω* = 3 and *σ* = −1, highlights that electronic charge density is located within a chemical bond between the involved atoms. Such a point is named a bond critical point (BCP).

## 4. Conclusions

The synthesis and the characterization of the asplatin prodrug and the study of its encapsulation into PSC4 to form a supramolecular adduct have been reported. The data reported in this work shows that the Pt(IV) complex can generate a supramolecular system with the PSC4 cavity, improving its cytotoxicity and biocompatibility profiles compared to free asplatin. The structural features of the system are well supported by the method of continuous variation (Job’s plot) and DFT analysis. The host-guest complex that involves the insertion of asplatin within the cavity of PSC4 is mainly stabilized by forming hydrogen bonds between the axial ligands of the platinum(IV) prodrug and a sulfonate moiety of the host cavity. The two most stable inclusion arrangements have been selected, and the corresponding estimated experimental and theoretical values of the free energy of complexation in solution are −6.8 kcal mol^–1^ and −13.2 kcal mol^–1^, respectively. Asplatin/PSC4 displayed stronger anticancer activities against MCF-7 as compared to oxaliplatin/PSC4 and carboplatin/PSC4 complexes. Additionally, the asplatin/PSC4 complex showed a more potent anticancer activity than the carboplatin/PSC4 complex against the A-549 cancer cell line. These findings demonstrate the promising potential of the asplatin/PSC4 complex as a biocompatible chemotherapeutic agent and also support the use of PSC4 as a delivery system for metal-containing chemotherapeutics and as a scaffold for designing novel carriers for antitumor prodrugs.

## Figures and Tables

**Table 1:** Mass spectrometry and elemental analyses of asplatin (C_9_H_14_Cl_2_N_2_O_5_Pt).

Mass spectrometry		M^+2^: 501.99 *m*/*z* (2.2%), M^+1^: 498.99 *m*/*z* (4.6%)
Elemental analysis	Calculated	C 21.64; H, 2.80; Cl 14.02; N 5.61; O 16.03; Pt 39.07%
	Found	C 21.79; H, 2.84; Cl 14.29; N 5.65; O 16.12; Pt 39.32%

**Table 2:** In vitro anticancer activities of PSC4, cisplatin, asplatin, and asplatin/PSC4 against human skin fibroblast, MCF-7, HeLa, and A-549 cell lines ^a^.

Cells	In Vitro Anticancer Activity (IC_50_; µg/mL)			
	PSC4	Cisplatin	Asplatin	Asplatin/PSC4
Human skin fibroblasts	>300	1.20 ± 0.23	3.41 ± 0.47	>300
MCF-7	>300	5.47 ± 0.44	1.54 ± 0.34	0.75 ± 0.05
HeLa	>300	5.94 ± 0.36	5.05 ± 0.49	2.15 ± 0.28
A-549	>300	9.61 ± 1.01	3.91 ± 0.58	3.60 ± 0.32

^a^ The treatment period was 48 h. Each value represents the mean ± standard deviation of triplicate measurements.

**Table 3:** Comparison between the in vitro anticancer activities of oxaliplatin/PSC4, carboplatin/PSC4, and asplatin/PSC4 complexes against MCF-7 and A-549 cancer cell lines.

Cells	In Vitro Anticancer Activity (IC_50_; µg/mL)		
	Oxaliplatin/PSC4	Carboplatin/PSC4	Asplatin/PSC4
MCF-7	1.56 ± 0.07	4.3 ± 0.2	0.75 ± 0.05
A-549		5 ± 0.4	3.60 ± 0.32

## Data Availability

Data is contained within the article or supplementary material.

## Supplementary Materials

The following supporting information can be downloaded at: https://www.mdpi.com/article/10.3390/ph15020259/s1, Figure S1: ^1^H-NMR characterization of Asplatin/PSC4 Complex; Figure S2: Absorbance spectra of several mixtures containing serially increasing concentrations of asplatin (0.01–0.25 mM) and a fixed concentration of 0.2 mM PSC4; Figure S3: Concentration-dependent effects of cisplatin, Asplatin, and PSC4/Asplatin on the cellular viability of (A-C) human skin fibroblasts, (D-F) MCF-7 cells, (G-I) HeLa cells, and (J – L) A-549 cells, respectively (after 48 h of treatment). All experiments were carried out in triplicates, and the mean values were calculated. Error bars represent ± standard deviation.
