# Supplementary material for: Synthesis, Characterization and Host-Guest Complexation of Asplatin: Improved In Vitro Cytotoxicity and Biocompatibility as Compared to Cisplatin
Source: Pharmaceuticals (Basel). 2022 Feb 21;15(2):259. doi: 10.3390/ph15020259 (PMC8875750; doi:10.3390/ph15020259)
Supplement: Supplementary file 1 [file pharmaceuticals-15-00259-s001.zip › pharmaceuticals-1560409-supplementary.pdf]

Supplementary Materials

Synthesis, Characterization and Host-Guest Complexation of Asplatin: Improved *In-vitro* Cytotoxicity and Biocompatibility As Compared to Cisplatin

Sherif Ashraf Fahmy[1,2], Fortuna Ponte[3], Giulia Grande[3], Iten M. Fawzy[4], Asmaa A. Mandour[4], Emilia Sicilia[3], Hassan Mohamed El-Said Azzazy[1,*]

1. Department of Chemistry, School of Sciences & Engineering, The American University in Cairo, AUC Avenue, PO. Box 74, New Cairo 11835, Egypt; sheriffahmy@aucegypt.edu
2. School of Life and Medical Sciences, University of Hertfordshire Hosted by Global Academic Foundation, R5 New Garden City, New Capital, Cairo 11835, Egypt
3. Department of Chemistry and Chemical Technologies, University of Calabria, Arcavacata di Rende, 87036, Italy; fortuna.ponte@unical.it; giulia-grande@hotmail.com; emilia.sicilia@unical.it
4. Pharmaceutical Chemistry Department, Faculty of Pharmacy, Future University in Egypt, Cairo 11835, Egypt; iten.mamdouh@fue.edu.eg; asmaa.abdelkereim@fue.edu.eg.

* Correspondence: hazzazy@aucegypt.edu, Tel.: +2 02 2615 2559; Fax: +2 02 2795 7565

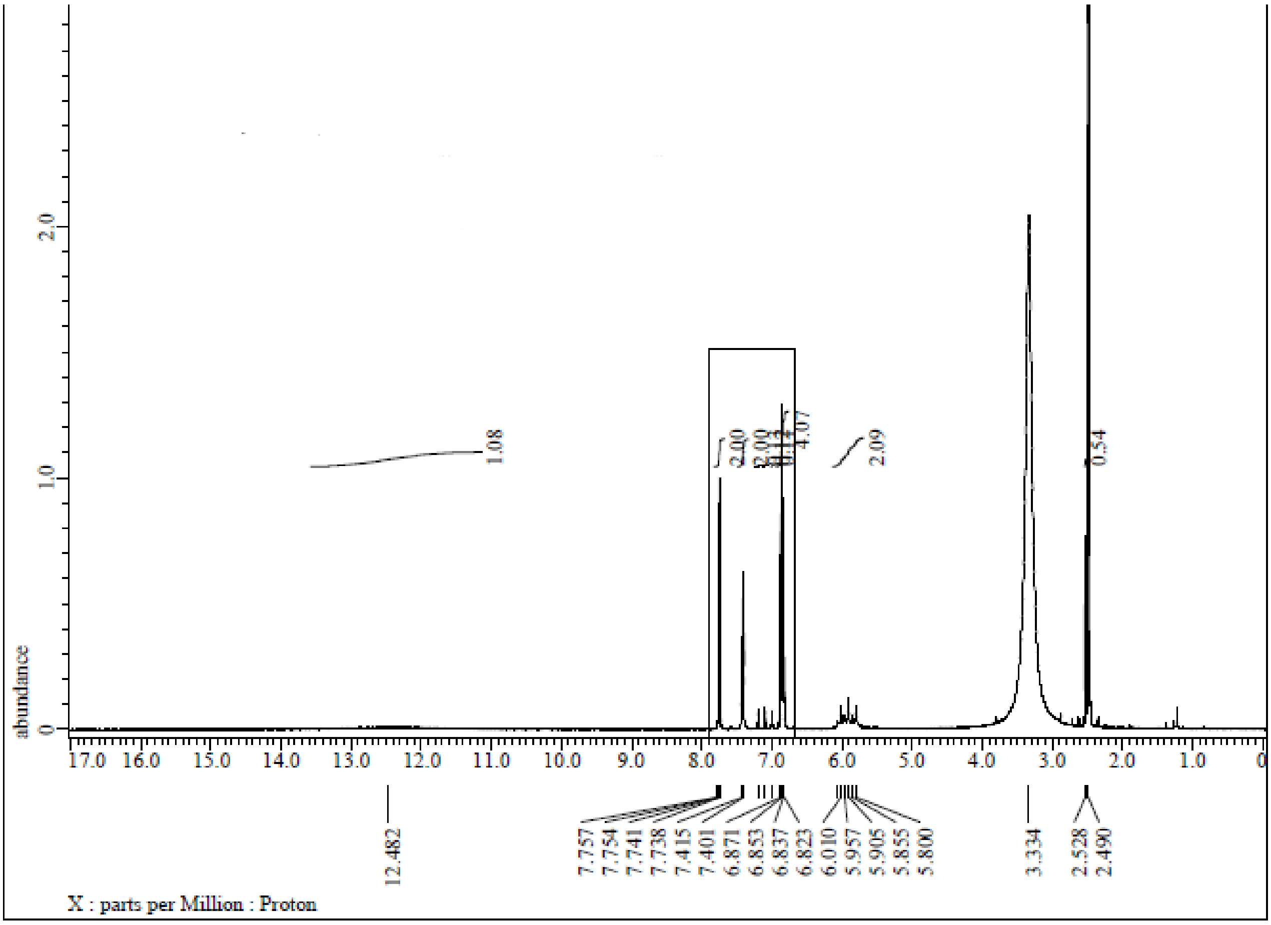

Figure S1. ^{1}H-NMR characterization of Asplatin/PSC4 Complex.

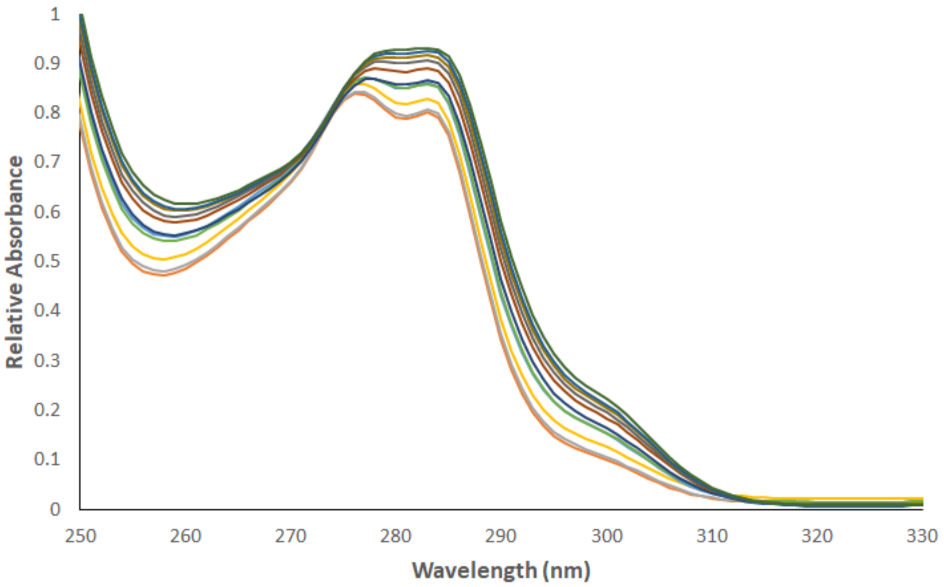

Figure S2. Absorbance spectra of several mixtures containing serially increasing concentrations of asplatin (0.01–0.25 mM) and a fixed concentration of 0.2 mM PSC4.

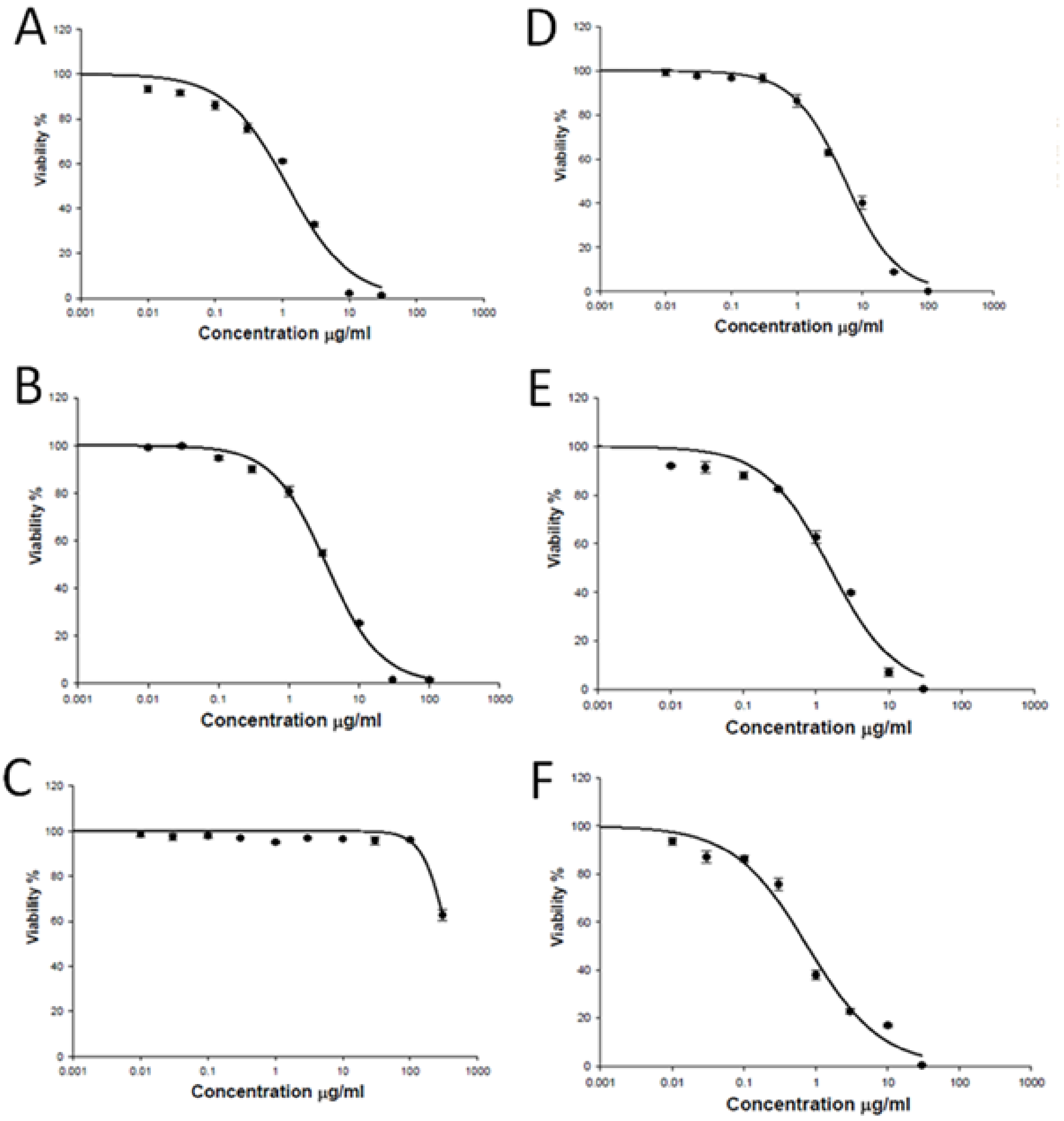
A
B
C
D
E
F
Viability %
Concentration µg/ml
0.001
0.01
0.1
1
10
100
1000
0
20
40
60
80
100
120

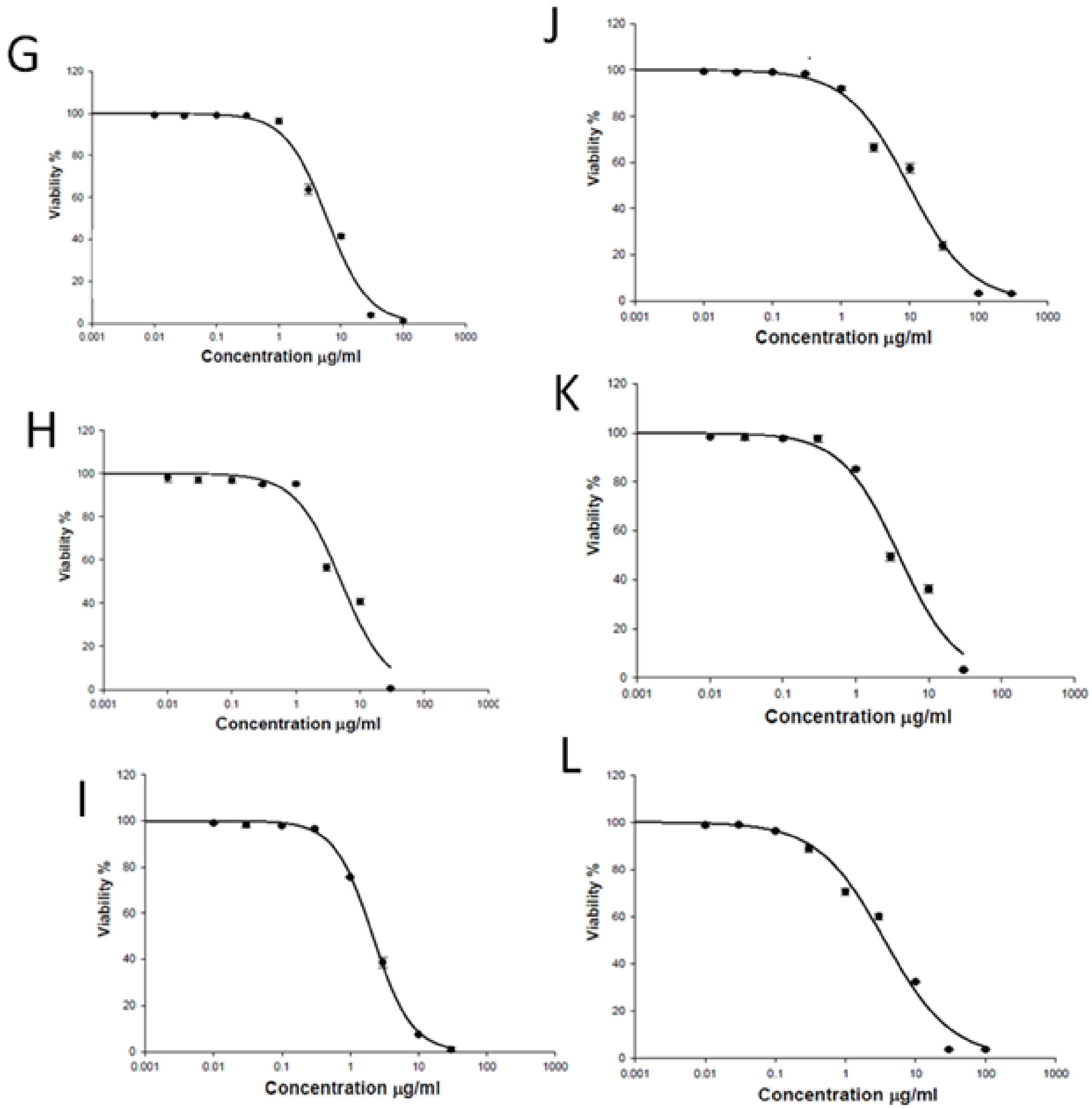

Figure S3. Concentration-dependent effects of cisplatin, Asplatin, and Asplatin/PSC4 on the cellular viability of (A-C) human skin fibroblasts, (D-F) MCF-7 cells, (G-I) HeLa cells, and (J – L) A-549 cells, respectively (after 48 h of treatment). All experiments were carried out in triplicates, and the mean values were calculated. Error bars represent ± standard deviation.
